# DEVELOPMENT OF SCAFFOLD FOR A ISCHEMIC STROKE MICROFLUIDIC CHIP PLATFORM

**DOI:** 10.1093/geroni/igae098.3649

**Published:** 2024-12-31

**Authors:** Lidadi Agbomi, Thomas Nathaniel, Richard Goodwin, Bruce Gao

**Affiliations:** Clemson University, Clemson, South Carolina, United States; University of South Carolina School of Medicine Greenville, Greenville, South Carolina, United States; University of South Carolina, Columbia, South Carolina, United States; Clemson University, Clemson, South Carolina, United States

## Abstract

Age-related brain diseases are rising, with strokes, especially ischemic strokes (IS), being a leading cause of death. Accurate modeling of IS is crucial for diagnosis, but animal models have limitations. When creating a new, human, cell-based microfluidic chip platform for modeling IS, a suitable 3D scaffold for cell growth is essential. For this, we chose to generate a 3D scaffold using a hydrogel platform that contains microchannels that will model the cerebral vasculature. 3D Agarose hydrogels were fabricated for testing. Hydrogels of 0.5%, 1%, and 2% agarose were polymerized according to the manufacturer’s protocol. Hydrogels were placed in a 60mm Petri dish. Testing was done on each hydrogel to determine the best agarose concentration after frozen for 10, 15, and 20 hours, then lyophilized for 8 hours. This method helped make the hydrogel more porous. The carbohydrate glass was fabricated by combining 53g of sucrose, 25g of glucose, 10g of Dextran, and 50 ml of Reverse Osmosis water and heated to 165°C. This solution was then poured into a heated 50mL glass syringe to be extruded into the hydrogel later. The 2% agarose hydrogel yielded the best results after lyophilization with the agarose maintaining some of its gel-like characteristics, while being more porous to help with fluid flow later. Preliminary tests were successful. Further tests will determine the best method for extruding carbohydrate glass into the hydrogel. Once it is extruded, the scaffold will be integrated into a microfluidic chip platform, and flow experiments will be initiated.

